# Hajdu-Cheney syndrome: a review

**DOI:** 10.1186/s13023-014-0200-y

**Published:** 2014-12-10

**Authors:** Ernesto Canalis, Stefano Zanotti

**Affiliations:** Departments of Orthopaedic Surgery and Medicine, UConn Health, 263 Farmington Avenue, Farmington, CT 06030 USA

**Keywords:** Notch, Skeleton, Bone remodeling, Hajdu-Cheney syndrome, Fractures, Polycystic kidneys, B cell lymphoma

## Abstract

Hajdu Cheney Syndrome (HCS), Orpha 955, is a rare disease characterized by acroosteolysis, severe osteoporosis, short stature, specific craniofacial features, wormian bones, neurological symptoms, cardiovascular defects and polycystic kidneys. HCS is rare and is inherited as autosomal dominant although many sporadic cases have been reported. HCS is associated with mutations in exon 34 of *NOTCH2* upstream the PEST domain that lead to the creation of a truncated and stable NOTCH2 protein with enhanced NOTCH2 signaling activity. Although the number of cases with *NOTCH2* mutations reported are limited, it would seem that the diagnosis of HCS can be established by sequence analysis of exon 34 of *NOTCH2*. Notch receptors are single-pass transmembrane proteins that determine cell fate, and play a critical role in skeletal development and homeostasis. Dysregulation of Notch signaling is associated with skeletal developmental disorders. There is limited information about the mechanisms of the bone loss and acroosteolysis in HCS making decisions regarding therapeutic intervention difficult. Bone antiresorptive and anabolic agents have been tried to treat the osteoporosis, but their benefit has not been established. In conclusion, Notch regulates skeletal development and bone remodeling, and gain-of-function mutations of *NOTCH2* are associated with HCS.

## Review

### Disease name/synonyms

Hajdu-Cheney syndrome; Acroosteolysis dominant type; Serpentine fibula polycystic kidney syndrome; Orpha number: 955

### Definition

Hajdu-Cheney syndrome (HCS) is a rare inherited connective tissue disease characterized by acroosteolysis of hands and feet, developmental defects of bones, teeth and joints causing distinctive craniofacial and skull changes, and also manifested by severe osteoporosis and short stature. The disease was first described by Hajdu in 1948 in a 37 year old accountant who died 12 years later of severe neurological complications, and the syndrome was reported further by Cheney in 1965 (Table 1) [1,2].

**Table 1:** **Hajdu**-**Cheney syndrome clinical features**

**Craniofacial features**	**Skeletal features**	**Other features**
• Facial dysmorphism, micrognathism	• Acroosteolysis, fibular deformities, fractures, joint hyperlaxity	• Short stature, developmental delay
• Open sutures, wormian bones	• Short and broad digits	• Polycystic kidneys
• Platybasia and basilar invagination	• Osteoporosis with fractures	• Neurologic symptoms, hearing loss
• Periodontal disease, tooth abnormalities and loss	• Vertebral deformities, scoliosis	• Congenital heart/vessel defect

### Epidemiology

HCS is a rare disease; less than 100 cases have been reported, but its exact prevalence is unknown.

### Clinical description

Hajdu-Cheney syndrome (HCS) is a rare disease with autosomal genetic inheritance, although the disease can also have sporadic presentations. It is characterized by acroosteolysis of distal phalanges, severe osteoporosis with fractures, craniofacial and dental abnormalities and short stature [1-7]. Patients with HCS exhibit prominent skeletal features including facial dysmorphisms, craniofacial defects, such as micrognathia, mid-face flattening and dental abnormalities. There is high clinical variability and a phenotypical evolution of the clinical manifestations. Some signs of the disease, such as synophrys, hypotelorism and epicanthal folds present as early as in the first two years of life and others become more evident in young children and adolescents, so that facial features become coarser over time [7]. Eventually, adult patients develop classic features of craniofacial dysmorphism characterized by bathrocephaly with prominent occiput, mild hypertelorism with telecanthus, downslanted eyes with synophrys, low-set ears, long philtrum, micrognathia with highly arched palate or cleft palate, and short neck. Acroosteolysis is frequently observed and can present with symptoms of inflammation, including pain and swelling. Patients have short and broad digits. Generalized and local joint hypermobility are reported frequently. Spinal abnormalities include compression fractures, deformities, kyphosis, scoliosis, platybasia and basilar invagination. Long bone deformities such as serpentine fibula also are noted [8]. Abnormal dental eruptions, decay and premature loss of teeth are common, and patients have a deep voice, hearing loss and hirsutism. Cardiovascular defects, including patent ductus arteriosus, atrial and ventricular septal defects, mitral and aortic valve abnormalities leading to valvular insufficiency or stenosis have been reported in HCS [9,10]. Respiratory infections can present in patients with HCS. Platybasia and basilar invagination are among the most serious complications of the disease and result in severe neurological problems, including hydrocephalus, central respiratory arrest and sudden death. Some patients present with renal cysts or polycystic kidneys, and serpentine fibula polycystic kidney syndrome seems to be the same disease as HCS.

### Aetiology

**Figure 1:**
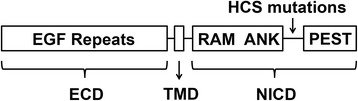
**Structure of NOTCH2 and mutations associated with Hajdu-** **Cheney syndrome.** The extracellular domain (ECD) of Notch consists of multiple epidermal growth factor (EGF) repeats, upstream the transmembrane domain (TMD). The intracellular domain of NOTCH2 (NICD) consists of a transcriptional domain formed by the Rbpjκ association module (RAM) linked to ankyrin (ANK) repeats, and nuclear localization sequences. The C-terminus contains the proline (P)-, glutamic acid (E)-, serine (S)-, and threonine (T)-rich motifs (PEST) domain which is required for the ubiquitinylation and degradation of the NICD. Nonsense and deletion mutations in exon 34 associated with Hajdu-Cheney syndrome (HCS) and pointed by the arrow lead to the formation of a truncated protein consisting of all NOTCH2 sequences necessary for the formation of the transcriptional complex, but lacking the PEST domain needed for the ubiquitinylation and degradation of NOTCH2. As such, a stable and active NOTCH2 protein is synthesized.

Over 60 years after the original description, whole exome sequencing of individuals affected by HCS revealed the presence of mutations in exon 34, the terminal exon of *NOTCH2*. Either nonsense mutations or deletions leading to a shift in the open reading frame and the creation of a termination codon in exon 34 of *NOTCH2* upstream the PEST domain are associated with HCS [11-14]. *NOTCH2* transcript levels are equivalent and not lower than those observed in controls, indicating a reduced capacity to activate the process of nonsense-mediated mRNA decay. This is common in mutations affecting terminal exons of a gene. Since the PEST domain contains sequences necessary for the ubiquitinylation and degradation of NOTCH2 in the proteasome, the mutations lead to the accumulation of a stable protein and persistence of NOTCH2 signaling since all sequences required for the formation of the Notch transcriptional complex are upstream the PEST domain and are therefore preserved (Figure 1). *NOTCH2* is located in Chromosome 1, 1p13 - p11.

It is of interest that somatic *NOTCH2* mutations causing loss of the PEST domain have been identified in B cell lymphoma, specifically in splenic marginal zone lymphoma [15-18]. These mutants exhibit enhanced transactivating activity in Notch reporter assays *in vitro* demonstrating that absence of the PEST domain leads to enhanced Notch activation [15,16]

Despite the pronounced skeletal abnormalities reported in HCS, little is known regarding the mechanisms underlying the bone loss. Although the distal phalangeal osteolytic lesions suggest increased localized bone resorption, there is no information on the mechanisms responsible for the generalized osteoporosis. The focal osteolysis is accompanied by neovascularization, inflammation and fibrosis [19-21]. Tissue from iliac crest biopsies has been examined in a small number of cases of HCS and revealed decreased trabecular bone, normal or increased bone remodeling, and normal or decreased bone formation [21-24]. In two published cases, increased number of osteoclasts with normal or increased osteoblasts were found suggesting that increased bone resorption may be responsible for the bone phenotype [21,25]. These observations are compatible with the known effects of Notch2 on the murine skeleton. In osteoclast precursors, Notch2 induces Nuclear factor of T-cells 1 transcription and osteoclastogenesis, and this effect could explain the increased bone remodeling [26,27]. Whether the osteoblast/osteocyte are also responsible for changes in bone turnover has not been established.

Mechanisms responsible for the craniofacial developmental abnormalities probably relate to the effects of Notch on skeletal development, and the short stature may be secondary to the inhibitory effects of Notch on chondrogenesis. Less is known about potential mechanisms to explain the periodontal disease and tooth loss and those responsible for the polycystic kidney disease. Notch plays a role in cardiovascular development and angiogenesis, and this would explain the congenital heart defects.

Missense mutations in exon 34 of *NOTCH2*, upstream of sequences encoding for the PEST domain, also have been detected in patients affected by serpentine fibula-polycystic kidney syndrome and the mutations are similar to those associated with HCS [4,28,29].

### Diagnosis

Patients with HCS have distinct radiologic findings, including acroosteolysis of distal phalanges of hands and feet. Plain radiographs of the skull reveal open sutures, intra sutural bones, abnormal flattening of the base of the skull, elongated sella turcica and absent frontal sinuses. Radiographs of the spine reveal bone loss and fractures. In addition, bone mineral density can be utilized to determine the presence of osteoporosis, although caution is required for its interpretation since diagnostic criteria established for postmenopausal osteoporosis may or may not apply. This is the case, for instance, with most forms of secondary osteoporosis, where a correlation between bone mineral density and fracture risk has not been established [30,31].

Based on a limited number of cases reported so far, the common pathogenetic mechanism in HCS seems to involve nonsense or deletion mutations in exon 34 of *NOTCH2*, resulting in a protein product lacking the PEST domain. Based on this information, the diagnosis of HCS would be carried out by sequence analysis of exon 34 of *NOTCH2*. For this purpose, genomic DNA is isolated from peripheral leukocytes and exon 34 amplified by polymerase chain reaction (PCR) using specific primers followed by sequence analysis of the PCR product [32].

### Differential diagnosis

HCS can present with a wide range of skeletal and non-skeletal manifestations so that the differential diagnosis may include a large array of clinical conditions. Acroosteolysis can be secondary to autoimmune disorders, such as scleroderma, systemic lupus erythematosus, Sjogren’s syndrome, rheumatoid arthritis and Raynaud’s disease; frostbite and injuries; neuropathies; diabetes mellitus; porphyria and psoriasis [19]. Osteoporosis, primary and secondary, should be considered in the differential diagnosis of HCS [33,34]. Werner’s syndrome and progeria, osteogenesis imperfecta and other rare skeletal disorders, such as Ehlers-Danlos syndrome, cleidocranial dysplasia, idiopathic juvenile osteoporosis also form part of the differential diagnosis [35].

### Genetic counseling and antenatal diagnosis

Most cases of HCS are sporadic, although in certain families autosomal dominant transmission is found [11-13].

There is limited information on genetic counseling and antenatal diagnosis regarding HCS. This is in part because the disease is rare, and because many cases of HCS are sporadic. In inherited cases, prenatal diagnosis could entail *NOTCH2* gene sequence analysis, although as indicated under Diagnosis, the number of HCS cases associated with *NOTCH2* mutations is limited and other gene mutations are possible. There is limited information about the penetrance of the disease, and it is conceivable that selected patients presenting with severe idiopathic osteoporosis are low penetrance cases of HCS.

### Management, including treatment of skeletal manifestations

The management of HCS requires a multi-system approach related to the organs affected by the disease in a given patient. Although patients develop acroosteolysis and osteoporosis, the mechanism of the bone loss is not known, making decisions regarding therapeutic interventions difficult. The acroosteolysis seems related to an inflammatory process. There are no controlled trials on the management of the osteoporosis; only anecdotal cases treated with either bisphosphonates or teriparatide. Bisphosphonate therapy (alendronate and pamidronate) alone or in combination with anabolic therapy with teriparatide has been attempted for the treatment of the skeletal manifestations of patients with HCS, but there is no clear evidence that either therapy is beneficial [36,37]. Teriparatide recently was shown to increase bone mineral density in a patient with HCS, but whether bisphosphonates or teriparatide offer fracture protection is not known [7]. Importantly, long-term activation of Notch signaling causes osteosarcoma in experimental mouse models, a potential concern when considering the use of teriparatide [38].

There is reasonable evidence indicating that activation of NOTCH2 signaling causes HCS, and NOTCH2 itself could be a future target for the treatment of the disease. Experimental modalities to control Notch signaling have been reported, including the use of antibodies to the Notch extracellular domain or its ligands, and the use of cell membrane permeable peptides that interfere with the formation of the Notch transcriptional complex [39,40]. These approaches could form the basis for the development of future therapies for HCS. However, reduced Notch signaling can result in the formation of vascular tumors in experimental animals [41]. There have been no studies reported in humans exploring these therapeutic approaches to block NOTCH2 signaling [41].

### Prognosis

HCS is a serious disease, but there is limited information about the overall prognosis of affected patients, and the prognosis is dependent on the organs affected and the complications of the disease. The natural evolution of HCS leads to the development of acroosteolysis and osteoporosis with fractures. As a consequence of the fractures, morbidity and mortality are increased [42]. The prognosis of patients with HCS is not favorable when there is evidence of neurological impairment. Basilar invagination is one of the most serious complications of HCS, and can occur in about 50% of the cases and result in neurological complications including central respiratory arrest [6]. Due to the limited number of cases, it is not possible to know whether lifespan is reduced, although it is reasonable to believe that patients with severe neurological complications may suffer from reduced lifespan.

### Molecular basis

Bone remodeling consists in the coordinated resorption and formation of bone, a process that requires the integrated involvement of cells of the osteoclast and osteoblast lineage and of signals released by these cells [43-48]. Osteoclasts are multinucleated cells derived from the fusion of mononuclear precursors of the hematopoietic lineage. The formation of osteoclasts requires receptor activator of nuclear factor κ-B ligand (RANKL) and macrophage colony stimulating factor (M-CSF) [49]. Osteoblasts are cells of mesenchymal origin, and their differentiation is tightly regulated by specific signals [50-53]. As osteoblasts become differentiated, they encounter various terminal destinies, including becoming embedded in distinct lacunae in the bone matrix as osteocytes, cells that play a fundamental role in mechanotransduction [54].

**Figure 2:**
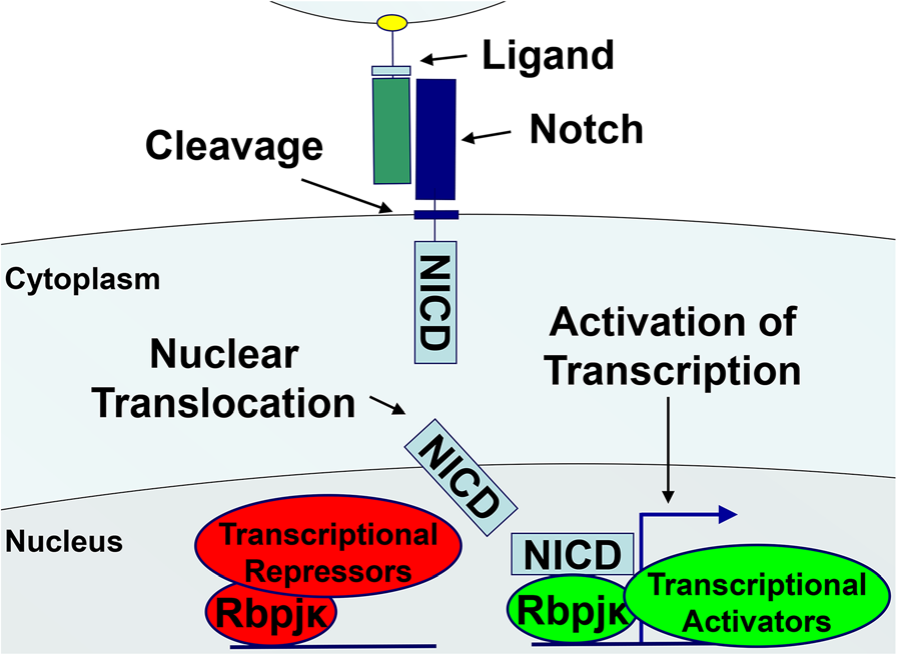
**Activation of Notch signaling.** Notch receptors and Jagged/Delta ligands are expressed as single-pass transmembrane proteins. Receptor-ligand interactions lead to the cleavage of the Notch receptor and release of the Notch intracellular domain (NICD) to the cytoplasm. NICD translocates to the nucleus and forms a ternary complex with Rbpjκ and Mastermind-like, displacing transcriptional repressors and associating with transcriptional activators, and inducing expression of Notch target genes.

Notch 1 to 4 are single-pass transmembrane receptors that play a critical role in cell fate decisions [55-60]. Notch has a complex structure, and its extracellular domain contains multiple epidermal growth factor-like tandem repeats upstream a negative regulatory region. Downstream the transmembrane domain, there is a Notch intracellular domain (NICD) consisting of an RBPJκ association module linked to ankyrin repeats, and together they form the Notch transcriptional domain. The C terminus contains the Proline (P), glutamic acid (E), serine (S) and threonine (T) rich (PEST) domain which is required for the ubiquitinylation and degradation of the NICD (Figure 1). There are five classic Notch ligands, which are Jagged1 and 2, and Delta Like1, 3 and 4 [61]. Notch-ligand interactions result in the proteolytic cleavage and release of the NICD, which translocates to the nucleus and interacts with Rbpjκ, and with Mastermind-like proteins to regulate transcription (Figure 2) [62-65]. This is termed the canonical signaling pathway, which leads to the transcription of Hairy and enhancer of split (Hes) 1, 5 and 7 and Hes-related with YRPW motif (Hey) 1, 2 and L [66]. The Notch non-canonical signaling pathway does not require Rbpjκ [61,67].

Skeletal cells express Notch1, Notch2 and low levels of Notch3 transcripts [68-70]. Notch regulates cell renewal in multiple organs and cell systems, and it is involved in skeletal development and homeostasis, and in osteoblast and osteoclast differentiation [57,61,71,72]. Transgenic overexpression of Notch1 NICD in cells of the osteoblastic lineage impairs osteoblast differentiation/function and causes osteopenia [72]. Notch signaling also suppresses chondrogenesis [57,73-80]. The effects of Notch in cells of the osteoblastic lineage are cell-context dependent and determined by the degree of differentiation of the cells targeted by Notch. When Notch is activated in undifferentiated cells of the osteoblastic lineage, it suppresses their progression to maturity and inhibits osteoblast function leading to the suppression of bone formation and as a consequence to bone loss [81]. In contrast, activation of Notch in mature osteoblasts and in osteocytes increases trabecular bone mass due to suppressed osteoclast formation and decreased bone resorption [81,82]. Accordingly, developmental or post-natal inactivation of *Notch1* and *Notch2* in osteoblast progenitors enhances cancellous bone volume by increasing osteoblast number and activity [57,83].

Most of the studies reported on the function of Notch in the skeleton have examined Notch1, so that less is known regarding the function of Notch2, 3 and 4. Notch1 and Notch 2 retain structural similarity, but these receptors have distinct activities, and global null mutations of either receptor result in embryonic lethality, indicating that they do not have redundant functions [84-87]. In contrast to the inhibitory effects of Notch1 on osteoclastogenesis, Notch2 enhances osteoclastogenesis [27,88].

### Unresolved questions

Current evidence indicates an association between mutations in Exon 34 of *NOTCH2* and HCS. However, the exact mechanisms by which NOTCH2 causes the manifestations of HCS are not known. Limited bone histological analysis has resulted in inconclusive results. It is not established whether the bone loss is the result of increased bone resorption, decreased bone formation or both. As a consequence, it is difficult to make optimal therapeutic decisions, and it is not known whether anti-resorptive and anabolic therapy will reduce the incidence of fractures in patients affected by the disease. Specific inactivation of NOTCH2 signaling by use of anti-NOTCH2 antibodies or soluble peptides that interfere with the formation of the transcriptional complex may offer novel alternative treatments. However, appropriate clinical trials are necessary to establish their effectiveness and potential adverse event profile. The pathogenesis of the acroosteolysis may be inflammatory leading to localized bone resorption but necessary studies to establish mechanisms involved are lacking and need to be conducted.

Mouse models of HCS, where mutations are introduced in Exon 34 of *Notch2* upstream the PEST domain should serve to study the disease, explore mechanisms involved and ways to reverse phenotypic manifestations. These should form the basis for a better understanding of the disease.

## Conclusions

Genetic mutations causing either gain- or loss-of-function of various components of the Notch signaling pathway are associated with diverse skeletal disorders, confirming that Notch is critical for skeletal development and homeostasis. Findings in human diseases are consistent with results of numerous pre-clinical studies. Although HCS affects a limited number of individuals, discovering a cluster of mutations in a single domain of *NOTCH2* in patients with HCS has advanced our knowledge regarding potential mechanisms leading to bone loss.

In conclusion, Notch signaling is required for skeletal development and bone homeostasis and diseases associated with dysregulation of Notch signaling are uncommon, but present with severe clinical manifestations.

## Abbreviations

ANK: Ankyrin
ECD: Extracellular domain
EGF: Epidermal growth factor
HCS: Hajdu-Cheney syndrome
*Hes*: Hairy enhancer of split
*Hey*: Hes-related with YRPW-motif
M-CSF: Macrophage colony stimulating factor
NICD: Notch intracellular domain
PEST: Proline (P), glutamic acid (E), serine (S) and threonine (T) rich
PCR: Polymerase chain reaction
RAM: Rbpjκ association module
RANKL: Receptor activator of nuclear factor κ-B ligand
TMD: Transmembrane domain
